# An Experimental Essay on the Physiology of the Blood

**Published:** 1839-01-01

**Authors:** 


### An Experimental Essay on the Physiology of the Blood. By *Charles Maitland*, M.D.

To this Essay was awarded the Gold Medal, by the Medical Faculty of Edinburgh—and it was well adjudged. The Essay is small (90 pages), but it contains a prodigious mass of facts and observations concentrated from all quarters, at home and abroad. Such a work is, of course, insusceptible of analysis—every page of it being a most concentrated analysis of memoirs that have preceded it. Nevertheless we do not like to pass it over, even with the warmest encomium, and without glancing at some of its multifarious contents.

In this Essay the author embraces only the physiology of the blood—its pathology being reserved for mature observation and experiments. It is thrown into five chapters, with a short introduction, containing the rise, progress, and downfall of the humoral pathology—on solidism—and on the present state of the question. Like most men of sense and experience, Dr. M. inclines to the opinion that solidism went as far in one extreme of error, as the humoral doctrine did in the other—much farther indeed. The blood is a *structure* circulating through all other structures—representing their wants, and diffusing the vital principle which it receives in the lungs, to every atom in the composition of the body. The following seems to us to be original and ingenious.

"The structures in the interior of our frame must have access to oxygen, and as they cannot be brought into contact with the atmosphere, some means must be devised for conveying oxygen to them. It is clear that the blood does not need the vast amount of that gas absorbed by it, for it is as constantly deprived of it in the extreme capillaries, and as the circulation is much slower in the veins than in the arteries, the blood must exist in the dark form during the greater part of our lives. We are, therefore, at no loss to ascertain by what means this element is furnished to the solids, and how they must suffer if this function be materially deranged. In this view, respiration is a general function of the system, deputed to the blood as by proxy, and performed in the pulmonary tissue; and the globules of the blood may be considered as representatives of the solids, by which these latter maintain their connexion with the seat of such important agencies." 5.

The blood, in the larger vessels, seems to have little association with the contiguous parts; but in the capillaries it is very different. When viewed with the microscope in this situation, Professor Schultz has compared it to a whirlpool, from which particles are constantly detached to be lost in the solids, whilst others are as perpetually flying off from the latter, to be merged in the crimson torrent. M. Andral remarks that, " at their point of contact, the blood assumes the nature of the solid, and becomes organized; so that its vitality can be no longer doubted." We think there can be no doubt of its vitality, in whatever situation it is to be seen.

"From this we are prepared to find that the morbid states of either division of our system may be mutually productive of each other; so much so, as often to prevent us from deciding in which of the two they take their origin. Since, therefore, the blood intervenes between the processes of digestion and nutrition, as well as between those of absorption and secretion, we might suppose from direct reasoning, that the proximate causes of disease in the nutritive and secernent systems might be shared between the organs concerned in those operations, and the fluid upon which they act. The progress of organic chemistry is daily accumulating proofs of the truth of this view of the matter." 6.

CHAP. I. *Mechanical Constitution of the Blood.* The temperature ranges from 100 to 101—specific gravity 1·05 to 7—viewed by the microscope it presents numerous red globules floating in a nearly colourless fluid. In man, and mammalia, the globules are flattened circular discs—in the lower ranges they are elliptical—and in the lowest, they return to the circular form again. The blood of birds shews the highest temperature—next is that of the omnivora, including man—then the herbivora—and last, the cold-blooded animals. In the same order stands the ratio of the richness of the blood in red globules. But the fluid constituent of blood—the liquor sanguinis—forms, perhaps, the most important element of our frame. It is essentially concerned in most of the animal functions, excepting respiration. In cholera it is filtered into the intestines—it is effused on the serous membranes, and forms adhesions—upon the mucous surfaces forming false membranes—into wounds in the shape of coagulable lymph—furnishing the materials for nutrition and secretion, &c. &c. "This fluid has been procured by artificial means, namely, by filtering the blood of a frog a little diluted with water. We used white filtering-paper, first moistened. In the liquid thus obtained, no globules can be discovered by the microscope. In a few minutes a coagulum forms, gradually contracts, becomes whitish and fibrous, and has exactly the appearance of lymph." This plays an important part in the changes which the blood undergoes when drawn from a vein.

"The effect of alcohol on fresh blood is somewhat remarkable. I do not find it noticed in any work on the subject. On mixing bullock's blood with half its weight of cold strong alcohol, it remained fluid for some hours; and was at that time immediately coagulated by adding an equal bulk of additional spirit to the mixture. Numerous brown flocks were separated, leaving a colourless fluid supernatant. On repeating the experiment with human blood, the same result was obtained. The following will be probably found a true explanation: the first portion of alcohol was sufficient to destroy the contractile power of the fibrin, but the water of the blood rendered it so dilute as not to precipitate the albumen; this, however, was effected by the farther addition of alcohol." 13.

Chap. II. *Chemical Constitution of the Blood.* Many attempts have been made, with very moderate success, to analyze this complicated fluid. Of late, however, the experimenters have been tolerably unanimous—and perhaps have come tolerably near the truth. Lecanu has lately published an elaborate analysis, which may be taken as the standard of the present chemistry of the blood. In two specimens of healthy blood, the following constituents were found.

| | | |
|---|---|---|
| " Water | 780·145 | 785·590 |
| Fibrin | 2·100 | 3·565 |
| Hematosine | 133·000 | 119·626 |
| Albumen | 65·090 | 69·415 |
| Crystalline fat | 2·430 | 4·300 |
| Oily matter | 1·310 | 2·270 |
| Extractive | 1·790 | 1·920 |
| Albumen combined with soda | 1·265 | 2·010 |
| Alkaline salts | 8·370 | 7·304 |
| Earthy salts, iron | 2·100 | 1·414 |
| Loss | 2·400 | 2·586 |
| | 1000·000 | 1000·000." 21. |

The third chapter discusses the effects of respiration on the blood—that often investigated stumbling-block of chemists and physiologists! The following are the chief facts—perhaps the only ones—that are allowed on all hands:—

"1. Of the air received into the lungs, part of the oxygen is retained, and a nearly equal volume of carbonic acid returned in its place, together with a variable amount of aqueous vapour.

2. The portion of blood transmitted through the pulmonary arteries and veins, has lost the dark modena hue, and acquired a red tint called arterial; together with which it receives a power of supporting life, which it did not possess before.

3. A portion of nitrogen is absorbed, and another portion evolved, generally nearly equal, but not always so.

4. On removing a portion of venous blood from the body, and agitating it with atmospheric air, oxygen is absorbed, carbonic acid evolved, and the red colour of arterial blood produced." 46.

We must pass over the chapter on the vitality of the blood, as leading to no useful or practical purpose. It is, as we said before, just as much entitled to the term vitality as the bones or muscles.

The last chapter is rather a diffuse one—"on the organic relations of the blood"—but presents a good epitome of all that is known respecting the constituents of the blood. We shall conclude with one extract from this chapter.

" *Hematosine.* Our knowledge of this substance is as yet very incomplete. Our first and last acquaintance with it is in its perfect state, for we saw before that the red tint of the chyle is merely owing to hematosine absorbed by lymphatics. And as we do not know of any further purposes which it serves beyond the circulation, so we have reason to believe that it is intended to be somewhat of a fixture in the system, its loss being supplied with difficulty. With the view of ascertaining the comparative facility with which these principles are renewed, the following case was selected. A middle-aged woman, affected with chronic bronchitis, was bled repeatedly at short intervals, with the view of relieving general oppression. On one of these occasions I obtained the blood, which was thus constituted :

| | |
|---|---|
| Water . . . . . . . . . | 829·9 |
| Fibrin and nuclein . . . . | 6·3 |
| Hematosine . . . . . . . | 45·6 |
| Albumen and salts . . . . | 118·2 |
| | 1000·0 |

From this and other observations, I conclude that hematosine is the most difficult to be replaced of all the elements of the blood. To apply this practically :—

A severe attack of sea-sickness, though it may reduce a man to a shadow, seldom produces lasting inconvenience; in almost all cases a good appetite and active stomach restore the bulk and vigour of the system in a surprisingly short space of time. In cholera, the introduction of saline solutions into the veins suddenly restores for a time the animation and even spirits of the patient. But in cases of severe inflammation, as of the lungs, when 140 ounces of blood have been occasionally drawn, we know how long such patients require to recover strength, especially their colour and animal temperature. Yet all this is accomplished readily by the transfusion of blood from another individual, and as before shewn, the fibrin is not concerned in this vivifying influence. In sea-sickness and cholera, the red globules are not diminished in quantity, and the other parts of the blood are readily replaced. Hence we learn to substitute other means of depletion for venesection in cases where we apprehend difficulty in the restoration of strength during convalescence." 83.

It was before remarked by Dr. Maitland that menstruation occasions a considerable loss of hematosine—and that it is not unreasonable to suppose that a deficiency of this is a predisposing cause of amenorrhœa. He asks whether the benefit derived from iron in such cases is a mere coincidence? Mr. Jennings found a deficiency of hematosine in cases of amenorrhœa, and our author did the same. The analysis of blood, in such an instance, was as follows :—

| | |
|---|---|
| Water . . . . . . . . . . | 781·0* |
| Fibrin . . . . . . . . . | 5.7 |
| Hematosine . . . . . . . | 81·8 |
| Albumen and salts . . . . | 131.5 |
| | 1000·0 |

If the reader will turn to the analysis of healthy blood, a page or two back, he will be surprised at the difference between that and the amenorrhœal blood.

We take leave of Dr. M., hoping that he will prosecute his inquiries into the pathology of the blood with as much zeal and success as he has shewn in its physiology.

* This case offers a remarkable instance of deviation from the general amount of constituents—the only deficiency is that of iron—the sum of albumen and hematosine being regular.

